# Discovery of new *Toxoplasma gondii* antigenic proteins using a high throughput protein microarray approach screening sera of murine model infected orally with oocysts and tissue cysts

**DOI:** 10.1186/s13071-018-2934-1

**Published:** 2018-07-04

**Authors:** Mert Döşkaya, Li Liang, Aarti Jain, Hüseyin Can, Sultan Gülçe İz, Philip Louis Felgner, Aysu Değirmenci Döşkaya, David Huw Davies, Adnan Yüksel Gürüz

**Affiliations:** 1 Department of Parasitology, Vaccine Research and Development Laboratory, Ege University Faculty of Medicine, Bornova/İzmir, Turkey; 2 Department of Medicine, Division of Infectious Diseases, University of California Irvine, Irvine, California USA; 3 Department of Molecular Biology, Ege University Faculty of Sciences, Bornova/İzmir, Turkey; 4 Department of Bioengineering, Ege University Faculty of Engineering, Bornova/İzmir, Turkey

**Keywords:** *Toxoplasma gondii*, Oocysts, Sporozoite, Tissue cyst, Bradyzoite, Protein microarray, Antigen discovery, Vaccine, Diagnostics

## Abstract

**Background:**

*Toxoplasma gondii* is an obligate intracellular protozoan parasite that causes congenital toxoplasmosis, as well as other serious clinical presentations in immune compromised humans. The parasite has also been recently linked to behavioral diseases in humans and other mammalian hosts. New antigens are being evaluated to develop a diagnostic kit for the diagnosis of acute infection or a protective vaccine.

**Methods:**

In this study, we have focused on the discovery of new antigenic proteins from *T. gondii* genomic data using a high throughput protein microarray screening. To date, microarrays containing > 2870 candidate exon products of *T. gondii* have been probed with sera collected from patients with toxoplasmosis. Here, the protein microarrays are probed with well-characterized serum samples from animal models administered orally with oocysts or tissue cysts. The aim was to discover the antigens that overlap in the mouse profile with human antibody profiles published previously. For this, a reactive antigen list of 240 antigens recognized by murine IgG and IgM was identified using pooled sera from orally infected mice.

**Results:**

Analyses of screening data have identified plenty of antigens and showed strong immunogenicity in both mouse and human antibody profiles. Among them, ROP1, GRA2, GRA3, GRA4, GRA5, GRA6, GRA7, GRA8, GRA14, MIC1, MIC2 and MAG1 have shown strong immunogenicity and used as antigen in development of vaccines or serological diagnostic assays in previous studies.

**Conclusion:**

In addition to the above findings, ROP6, MIC12, SRS29A and SRS13 have shown strong immunogenicity but have not been tested in development of a diagnostic assay or a vaccine model yet.

**Electronic supplementary material:**

The online version of this article (10.1186/s13071-018-2934-1) contains supplementary material, which is available to authorized users.

## Background

*Toxoplasma gondii* is a protozoan parasite that has a widespread distribution worldwide among humans and animals [1]. The importance of this intracellular parasite comes from the clinical presentations formed in the fetus and in immune compromised patients. Infection of women early in pregnancy is associated with an increased risk of congenital toxoplasmosis *in utero* and may become lethal by dissemination to vital organs such as brain, heart or lungs. This may also occur in immunocompromised adults by therapeutic immunosuppression, such as in cancer chemotherapy or organ transplantation [1]. Recently, *T. gondii* has also been linked to behavioral syndromes such as schizophrenia or bipolar disorder [2].

The transmission of toxoplasmosis mainly occurs through contaminated water and foods [1]. Felidae cats are definitive hosts in which the production of oocysts occurs. Cats disseminate resistant oocysts in their feces that can be contagious up to 18 months in the environment. Humans, as well as other intermediate hosts, are often infected *via* domestic cats, but can also become infected by ingestion of tissue cysts in raw or under-cooked meat prepared from infected hosts.

Due to ease of transmission and resistance of oocysts in the environment, 1/3 of humans are estimated to be infected with *T. gondii* worldwide [3]. The rate of toxoplasmosis among pregnant women range between 37–58% in Europe [3], compared to 10.8% in the USA. [4]. In the USA, each year 400–4000 infants are estimated to be born with congenital toxoplasmosis, and up to 1.26 million cases of ocular toxoplasmosis are reported [3]. In addition, *T. gondii* oocysts are classified as a category B bioterrorism agents owing to a potential threat to water safety [5], as demonstrated by tainted water outbreaks that occurred in British Columbia, that affected 7718 people [6], Santa Isabel do Ivai of Brazil that affected hundreds of people [7], Coimbatore, India that affected 178 people [8], and Izmir that affected 171 Air Force Academy recruits [9].

In addition to being the definitive host for *T. gondii*, abundance and close proximity of cats as a companion species in human populations plays an important role in transmission of infections to humans. According to the American Veterinary Medical Association (AVMA) and the European pet food industry Federation (FEDIAF) the total number of domestic cats in the USA is over 74 million, or approximately 1 cat per family of 4, and 74 million in the European Union [10, 11].

During acute toxoplasmosis in pregnant women, fetus can get infected by *T. gondii* and congenital toxoplasmosis may occur. The first problem is misdiagnoses of acute toxoplasmosis in pregnant women during the first trimester due to the insufficiency of serological assays that cannot discriminate acute infection and chronic infection. Antigens specific for acute infection can be used in these serological assays for timely diagnosis of the disease. The other problem is that an efficient vaccine that can protect pregnant women against toxoplasmosis does not exist. Current diagnostic antigens that are used to develop diagnostic assays or vaccine candidate antigens are almost always selected randomly and/or based on biological properties such as being a surface protein, having a role in pathogenesis, or high immunogenicity [12, 13]. In this new era of advanced technology, methods to select antigens should specifically take advantage of recent advances such as *in silico* analysis and novel *in vitro* and *in vivo* immunoscreens for the generation of new antigens. The present study addresses this bottleneck in selection of antigenic proteins against toxoplasmosis using a high throughput protein microarray screening approach [14]. Initially, we have generated protein microarray chips containing 2870 candidate exon products of *T. gondii.* Then, we probed them with well grouped human sera [(i) acute (IgM- positive and low avidity IgG); (ii) chronic with IgM persistence (IgM-positive and high avidity IgG); (iii) chronic (IgM-negative and high avidity IgG); and toxoplasmosis negative] [15, 16]. There was a shortcoming in this approach that experimental infections are not possible in humans and we could not determine the antigens that were present at the beginning of the infection. This knowledge was important since these antigenic proteins can be very important to develop a diagnostic assay or a vaccine.

Following the two protein array studies in human toxoplasmosis, here we report a comprehensive *in vivo* study in a mouse model screened by protein microarrays. Mouse is an intermediate host with immune response similar to humans. For this purpose, two mouse groups were infected orally with oocysts and tissues cysts mimicking natural route of infection. Sera were collected prior to infection (Day 0) and at 1, 2, 3, 6, 10, 15, 40 and 120 days after infection. Then, protein microarray slides were probed with these sera to identify immunodominant exon products present in acute and chronic murine toxoplasmosis. Infecting the mouse groups with oocysts or tissue cysts also gave us the opportunity to determine the dominant antigens presented *via* the fecal-oral and ingestion routes of entry (i.e. mimicking natural route of infection).

## Methods

### Animals

Six to eight weeks-old female Swiss outbred mice were obtained from the Bornova Veterinary Control Institute Animal Production Facility. *Toxoplasma gondii* oocysts were obtained from a recently weaned kitten, approximately 3–4 months-old.

During *in vivo* studies, mice and cat were housed in different rooms under standard and suitable conditions. Specifically, rooms had ambient temperature and humidity, adequate light cycle, and diet was specific for each animal type. All animals were checked for humane endpoints every day such as rapid weight loss more than ~20% of gross body weight, inability to assess water or food, or loss of skin elasticity indicative of dehydration.

### Obtaining tissue cysts and oocysts

A kitten was fed with *T. gondii* PRU strain tissue cysts obtained from mouse brain as described [17]. Initially, brains of previously infected mice were extracted under sterile conditions and homogenized in approximately 2 ml sterile 0.9% NaCl containing penicillin (10 U/ml), streptomycin (10 μg/ml) and gentamicin (2 μg/ml) using an injector with 20G 1" (0.9 × 25 mm) needle. The tissue cysts were counted by hemocytometer under phase contrast microscope (Nikon) and the remaining homogenate was used to passage the strain *in vivo*.

To feed the kitten, a part of the infected mouse brain was crushed between cover glass and slide and tissue cysts were examined under phase contrast microscope. The remaining brain with tissue cysts was fed to the cat. Before feeding the kitten, the presence of oocysts in kitten stool was examined using sucrose flotation technique as described [17].

After feeding, the feces of the kitten were collected daily and oocysts were purified as described [17–19]. Briefly, ~10 g of feces were softened by adding to a 50 ml tube, filled with tap water and incubated for 2 h at room temperature. The tap water was discarded and approximately 50 ml sucrose/phenol solution (53 g sucrose, 0.8 ml liquid phenol, 100 ml tap water) was added to the softened feces, and slowly emulsified using a spatula. Next, the homogenate was filtered through two layers of sterile gauze and centrifuged at 400× *g* for 10 min. Then, 500 μl supernatant from the top of the tube was collected and mixed with 4.5 ml 2% H_2_SO_4_ to stimulate sporulation. The oocysts were then incubated at room temperature for 3–5 days and checked for sporulation by microscopy. As the oocysts sporulated, 3 ml of 1 N NaOH was added to the mixture to stop stimulation.

For purification of oocysts, 4 ml of 2.2 M sucrose solution was mixed with the sporulated oocysts and then 5 ml distilled H_2_O was carefully overlaid and centrifuged at 1200× *g* for 20 min. Supernatant containing oocysts was collected without disturbing the sucrose solution, and 4 ml of 2.2 M sucrose solution was mixed with the supernatant again. Then, 5 ml distilled H_2_O was carefully overlaid and centrifuged at 1200× *g* for 20 min. The supernatant containing oocysts was collected, resuspended in 50 ml of distilled water, and centrifuged at 2000× *g* for 10 min. About 1–2 ml of the supernatant collected from the top of the tube was mixed with an equal volume of sterile 0.9% NaCl. The resulting purified oocysts were quantified by hemocytometer under phase contrast microscopy and immediately used to infect mice.

### Infection of mice and collection of sera

Two groups of Swiss outbred mice (*n* = 10) were administered orally with fresh 8–10 sporulated oocysts and 10–15 tissue cysts using a stainless steel curved feeding needle (Harvard Apparatus). Serum samples were collected by tail bleeding from each anesthetized mouse prior to inoculation (day 0) and on day 1, 2, 3, 6, 10, 15, 40 and 120 after infection. On day 120, mice were euthanized and brains were homogenized in approximately 2 ml sterile 0.9% NaCl containing penicillin (10 U/ml), streptomycin (10 μg/ml) and gentamicin (2 μg/ml) using a syringe with a 20G 1" (0.9 × 25 mm) needle. The presence of tissue cysts in each homogenate was confirmed using a phase contrast microscopy to determine if mice were infected with *T. gondii*.

### Protein microarray fabrication to determine IgM and IgG kinetics

A two-step antigen discovery process was employed. In the first step, two large arrays (TG1 and TG2/3) comprising more than 2870 proteins were probed with a subset of the murine immune sera (sera from nine mice collected at day 10–15 and 40) to determine the IgG and IgM responsive predominant antigens. The first published array (TG1) comprised 1357 exon products from 615 genes [15] and the second array (TG2/3) contained 1513 exons (from 772 genes) [16]. These 2870 proteins were down-selected from > 8000 genes (> 43,000 exons) present in the *T. gondii* genome by a bioinformatic filtering process, as previously described [15, 16]. To isolate the exons, primers were designed from the genomic sequence of type II strain ME49 of *T. gondii* that was obtained from the *Toxoplasma* Genomics Resource (http://toxodb.org/toxo/). In the final screening, a completely new mouse array (TG4) was generated. Initially, a set of sera from day 40 for IgG and two sets of sera from day 10 and day 15 were probed against the full TG1 and TG2/3 arrays to determine the murine profile. Finally, a total of 240 exon products reactive to the murine sera were identified on the basis of this screen. These 240 plasmids were expressed and printed using the standard methods to produce a mouse-appropriate, down-selected reactive antigen array, termed here “TG4” (see Additional file 1: Table S1 for the list of 240 prioritized proteins). The final TG4 array was generated and probed with all the mouse sera samples (from day 0 to day 120) as described previously [15, 16]. Then, the results of the array were compared to the human array profiles to determine the shared antigens both in humans and mice.

The nomenclature used in ToxoDB *Toxoplasma* Genomic Resource (http://toxodb.org/toxo/) is used throughout, with each gene ID appended with the exon number expressed.

### Statistical analysis

The statistical analyses of arrays were handled as described previously [15, 16]. Briefly, raw spot and local background intensities, protein annotation, and sample phenotypes are imported and merged in the R statistical environment, where all subsequent procedures are performed unless specified otherwise. Microarray spot intensities are quantified using ScanArrayExpress software (Perkin Elmer, USA) utilizing automatic local background subtraction for each spot. The generated signal intensity values are considered raw values. Next, all raw values are transformed using the base 2 logarithm (log_2_). Antibody magnitude is defined as signal intensity of reactive antigens with respect to NoDNA probes. To calculate the magnitude of antibody responses, the median normalized signal intensity (log_2_ scale) of the NoDNA probes per individual is subtracted from target probe normalized signal intensities for each individual.

All statistical analyses for microarray data were performed using the R statistical environment software (http://www.r-project.org). A Bayes regularized t-test is adapted from Cyber-T for protein arrays. To account for multiple testing conditions, the Benjamini & Hochberg [20] (BH) method is used to control the false discovery rate. After Benjamini-Hochberg correction, *P*-values smaller than 0.05 are considered significant, and the corresponding protein is considered differentially reactive.

## Results

Serum samples were collected from two groups of mice, infected orally with viable oocysts and tissue cysts, before infection (Day 0) as well as 1, 2, 3, 6, 10, 15, 40 and 120 days after infection. Next, IgM and IgG antibody kinetics of these sera were analyzed by protein microarrays comprising 240 prioritized proteins.

### IgM kinetics of mice infected with oocysts

**Fig. 1:**
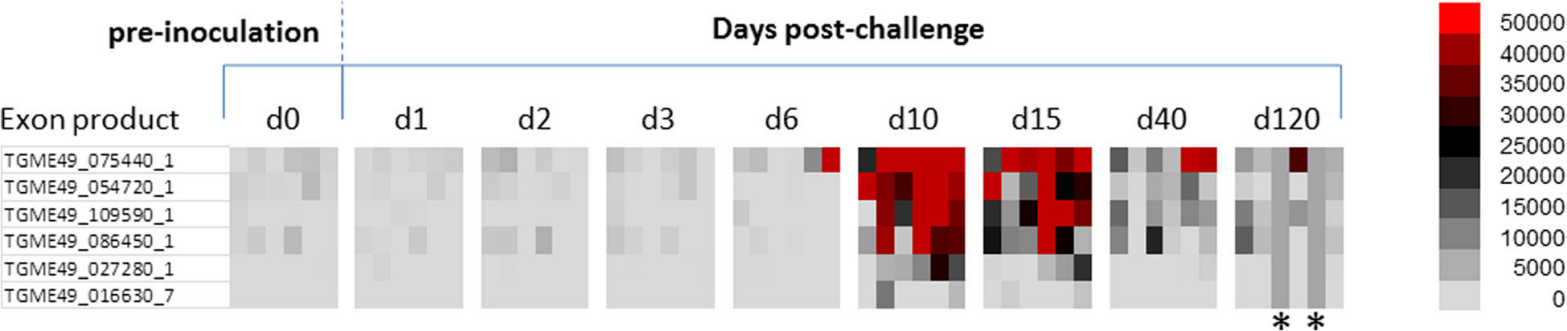
Heat map representing IgM profiles at different time points in mice inoculated orally with oocysts. According to the heat map, six antigens showed significant increase at day 10 post-infection compared to day 0 pre-inoculation values. Grey columns indicated with * show the mice that did not survive until day 120

**Fig. 2:**
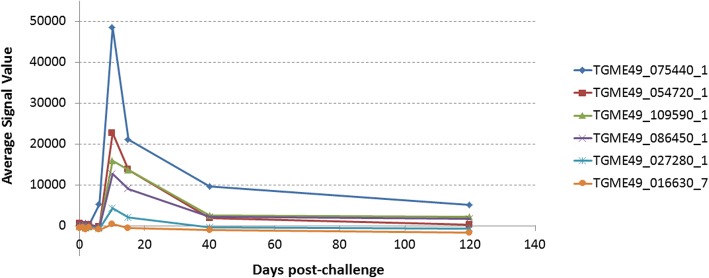
Line chart of IgM kinetics at different time points in mice inoculated orally with oocysts. Line chart of six antigens, shown in Table 1 overlaid with Benjamini-Hochberg-corrected *P*-values comparing data for day 10 post-infection with pre-inoculation values. Signal values are average of adjusted array signals (raw *in vitro* transcription/translation [IVTT] signals subtracted of median of sample-specific control IVTT spots) ± SD

During the screening in the group of mice infected with viable oocysts, the IgM response reached maximal intensity and breadth around day 10, as seen in the heat map in Fig. 1. Responses against 6 antigens increased significantly at day 10 compared to day 0 (*P*-values are listed in Table 1). Of the 5 antigens listed in Table 1, one of them is rhoptry protein and four are dense granule proteins which were all excreted secreted antigens (ESA) (Table 1). The IgM response decreased steadily after day 10 (Fig. 2).

**Table 1:** Antigens that showed significant IgM response at day 10 in mice infected with oocysts

Rank ^a^	Gene ID_exon	Description	Benjamini-Hochberg corrected *P*-value (d0 *vs* d10)
1	TGME49_075440_1	Granule antigen protein GRA6	*P* < 0.0001
2	TGME49_054720_1	Dense granule protein GRA8	*P* < 0.0001
3	TGME49_109590_1	Rhoptry protein 1	*P* = 0.0007
4	TGME49_086450_1	Dense granule protein 5 precursor	*P* = 0.0131
5	TGME49_027280_1	Dense granule protein GRA3	*P* = 0.0077
6	TGME49_016630_7	Trigger factor protein, putative	*P* = 0.0183

^a^Based on average signal value, as per Figs. 1 and 2

### IgG kinetics of mice infected with oocysts

**Fig. 3:**
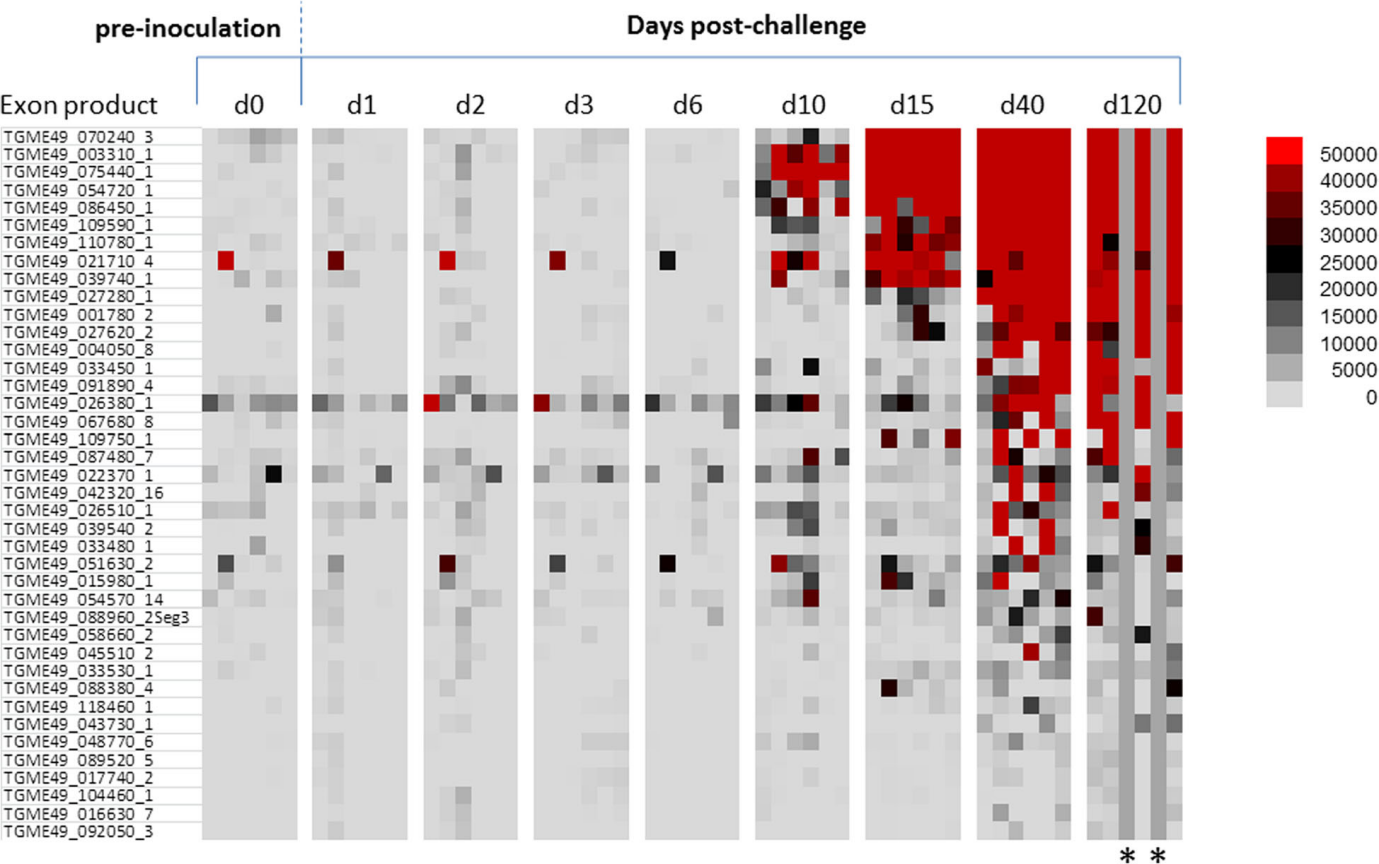
Heat map representing IgG profiles at different time points in mice inoculated orally with oocysts. According to the heat map, 40 antigens showed significant increase at day 40 post-infection compared to day 0 pre-inoculation values. Grey columns indicated with * represent the mice that did not survive until day 120

**Fig. 4:**
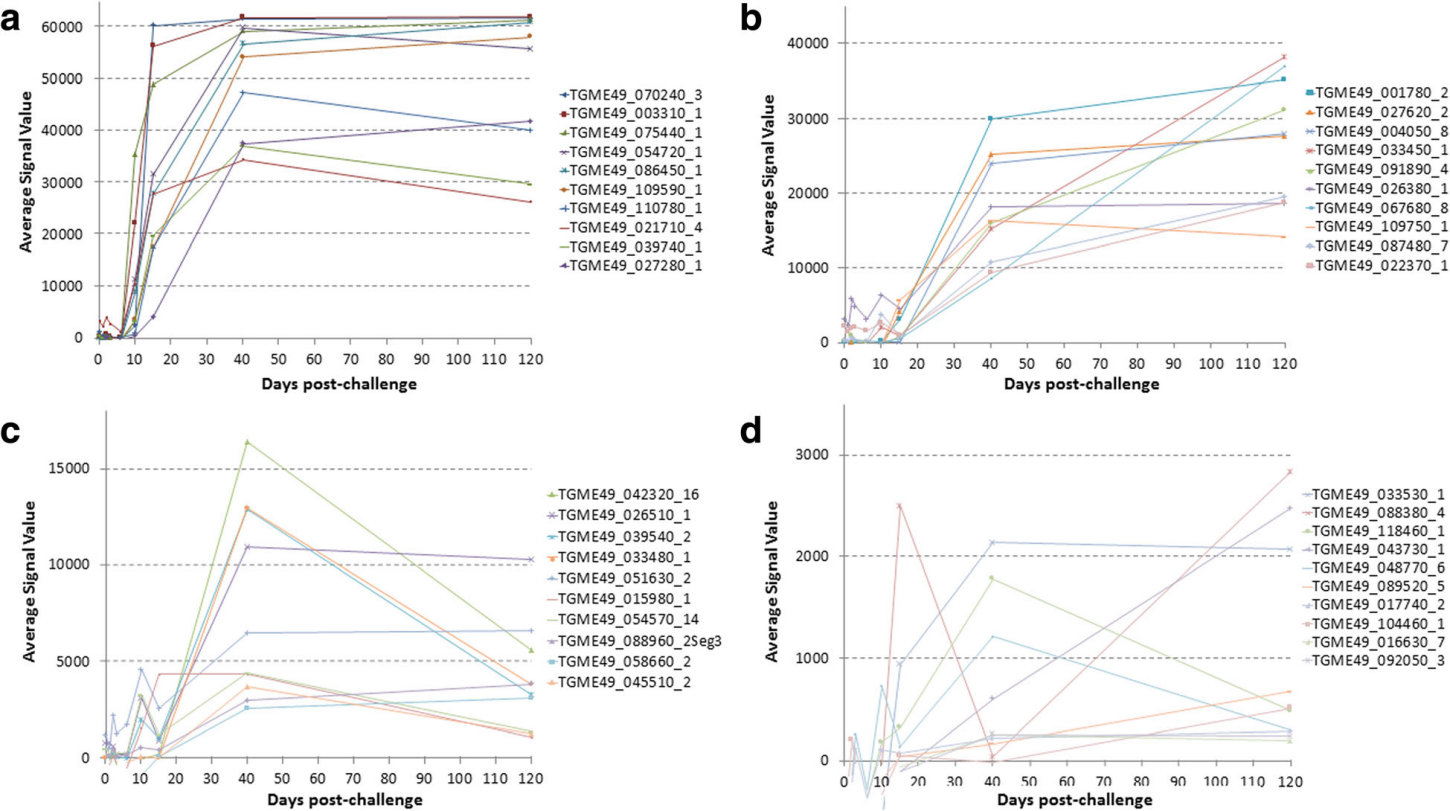
Line charts show IgG kinetics of 40 antigens obtained from sera of mice inoculated orally with oocysts. Serum samples were collected from each mouse prior to inoculation (day 0) and on day 1, 2, 3, 6, 10, 15, 40 and 120 after infection. The antigens in line chart are also shown in Table 2 overlaid with Benjamini-Hochberg-corrected *P*-values which compares the absorbance average signal value data for day 40 post-infection with day 0 pre-inoculation values. In order to increase the visibility, line charts of 40 antigens are shown in four consecutive panels (**a**-**d**). Signal values are average of adjusted array signals (raw IVTT signals subtracted of median of sample-specific control IVTT spots) ± SD

During the screening, IgG response against 40 antigens reached maximal intensity and breadth around day 40 as seen in the heat map in Fig. 3. IgG responses against 40 antigens increased significantly at day 40 compared to day 0 (*P*-values are listed in Table 2). The IgG response mostly first peaked at day 10 or afterwards (Fig. 4a-d). Among the first five highest signal giving proteins, four of them are dense granule proteins which were ESA (Table 2).

**Table 2:** Antigens that showed significant IgG response at day 40 in mice infected with oocysts

Rank ^a^	Gene ID_exon	Description	Benjamini-Hochberg corrected *P*-value (d0 *vs* d40)
1	TGME49_070240_3	Unspecified product (MAG1)	*P* < 0.0001
2	TGME49_003310_1	Dense granule protein GRA7	*P* < 0.0001
3	TGME49_075440_1	Granule antigen protein GRA6	*P* < 0.0001
4	TGME49_054720_1	Dense granule protein GRA8	*P* < 0.0001
5	TGME49_086450_1	Dense granule protein 5 precursor	*P* < 0.0001
6	TGME49_109590_1	Rhoptry protein 1	*P* < 0.0001
7	TGME49_110780_1	Dense granule protein GRA4	*P* < 0.0001
8	TGME49_021710_4	TBC domain-containing protein	*P* = 0.0014
9	TGME49_039740_1	Dense granule protein GRA14	*P* < 0.0001
10	TGME49_027280_1	Dense granule protein GRA3	*P* < 0.0001
11	TGME49_001780_2	Microneme protein MIC2	*P* = 0.0003
12	TGME49_027620_2	Dense granule protein GRA2	*P* < 0.0001
13	TGME49_004050_8	Subtilisin SUB1	*P* = 0.0007
14	TGME49_033450_1	SAG-related sequence SRS29A	*P* = 0.0151
15	TGME49_091890_4	Microneme protein MIC1	*P* = 0.0003
16	TGME49_026380_1	Hypothetical protein	*P* = 0.0429
17	TGME49_067680_8	Microneme protein MIC12	*P* = 0.0151
18	TGME49_109750_1	Succinate-Coenzyme A ligase, beta subunit	*P* = 0.0275
19	TGME49_087480_7	Hypothetical protein	*P* = 0.0483
20	TGME49_022370_1	SAG-related sequence SRS13	*P* = 0.0445
21	TGME49_042320_16	B-box zinc finger domain-containing protein	*P* = 0.0190
22	TGME49_026510_1	Sec23/Sec24 trunk domain-containing protein	*P* = 0.0359
23	TGME49_039540_2	LEM3 (ligand-effect modulator 3) family	*P* = 0.0280
24	TGME49_033480_1	SAG-related sequence SRS29C	*P* = 0.0358
25	TGME49_051630_2	Slc30a2 protein	*P* = 0.0361
26	TGME49_015980_1	Hypothetical protein	*P* = 0.0358
27	TGME49_054570_14	Hypothetical protein	*P* = 0.0483
28	TGME49_088960_2Seg3	Hypothetical protein	*P* = 0.0358
29	TGME49_058660_2	Rhoptry protein ROP6	*P* = 0.0358
30	TGME49_045510_2	Phospholipid-translocating P-type ATPase	*P* = 0.0483
31	TGME49_033530_1	Hypothetical protein	*P* = 0.0364
32	TGME49_088380_4	Heat shock protein HSP90	*P* = 0.0430
33	TGME49_118460_1	P-type ATPase	*P* = 0.0430
34	TGME49_043730_1	Rhoptry protein ROP9	*P* = 0.0364
35	TGME49_048770_6	Hypothetical protein	*P* = 0.0430
36	TGME49_089520_5	Hypothetical protein	*P* = 0.0498
37	TGME49_017740_2	3-ketoacyl-(acyl-carrier-protein) reductase	*P* = 0.0483
38	TGME49_104460_1	Zinc finger, C3HC4 type (RING finger) domain-containing protein	*P* = 0.0483
39	TGME49_016630_7	Trigger factor protein, putative	*P* = 0.0498
40	TGME49_092050_3	Calcium dependent protein kinase CDPK8	*P* = 0.0483

^a^Based on average signal value, as per Figs. 3 and 4

### IgM kinetics of mice infected with tissue cysts

**Fig. 5:**
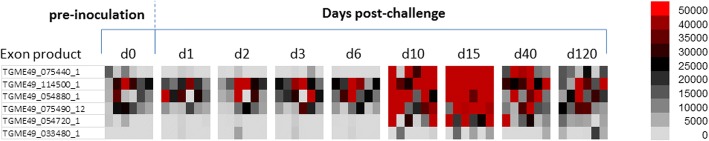
Heat map representing IgM profiles at different time points in mice inoculated orally with tissue cysts. According to the heat map, six antigens showed significant increase at day 10 post-infection compared to day 0 pre-inoculation values

**Fig. 6:**
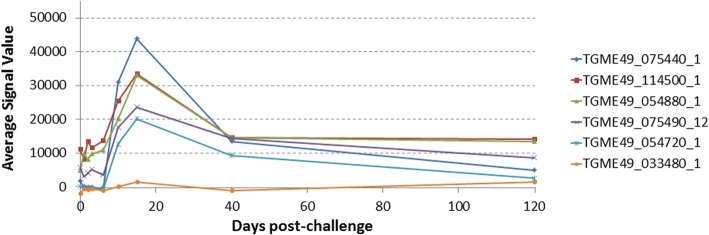
Line chart represent IgM kinetics specific for six antigens probed with sera collected from mice at different time points inoculated orally with tissue cysts. These six antigens are also shown in Table 3 overlaid with Benjamini-Hochberg-corrected *P*-values comparing the absorbance average signal value data for day 10 post-infection with day 0 pre-inoculation values. Signal values are average of adjusted array signals (raw IVTT signals subtracted of median of sample-specific control IVTT spots) ± SD

During the screening in the group of mice infected with viable tissue cysts, the IgM response reached maximal intensity and breadth around day 10, as seen in the heat map in Fig. 5. IgM responses against 6 antigens increased significantly at day 10 compared to day 0 (*P*-values are listed in Table 3). Of the 5 antigens listed in Table 1, one of them is rhoptry protein and two of them are dense granule proteins which all ESA. The IgM response decreased steadily after day 15 (Fig. 6).

**Table 3:** Antigens that showed significant IgM response at day 10 in mice infected with tissue cysts

Rank ^a^	Gene ID_exon	Description	Benjamini-Hochberg corrected *P*-value (d0 *vs* d10)
1	TGME49_075440_1	Granule antigen protein GRA6	*P* = 0.0493
2	TGME49_114500_1	Subtilisin SUB2	*P* = 0.0497
3	TGME49_054880_1	Alpha-galactosidase	*P* = 0.0493
4	TGME49_075490_12	Hypothetical protein	*P* = 0.0493
5	TGME49_054720_1	Dense granule protein GRA8	*P* = 0.0493
6	TGME49_033480_1	SAG-related sequence SRS29C	*P* = 0.0493

^a^Based on average signal value, as per Figs. 5 and 6

### IgG kinetics of mice infected with tissue cysts

**Fig. 7:**
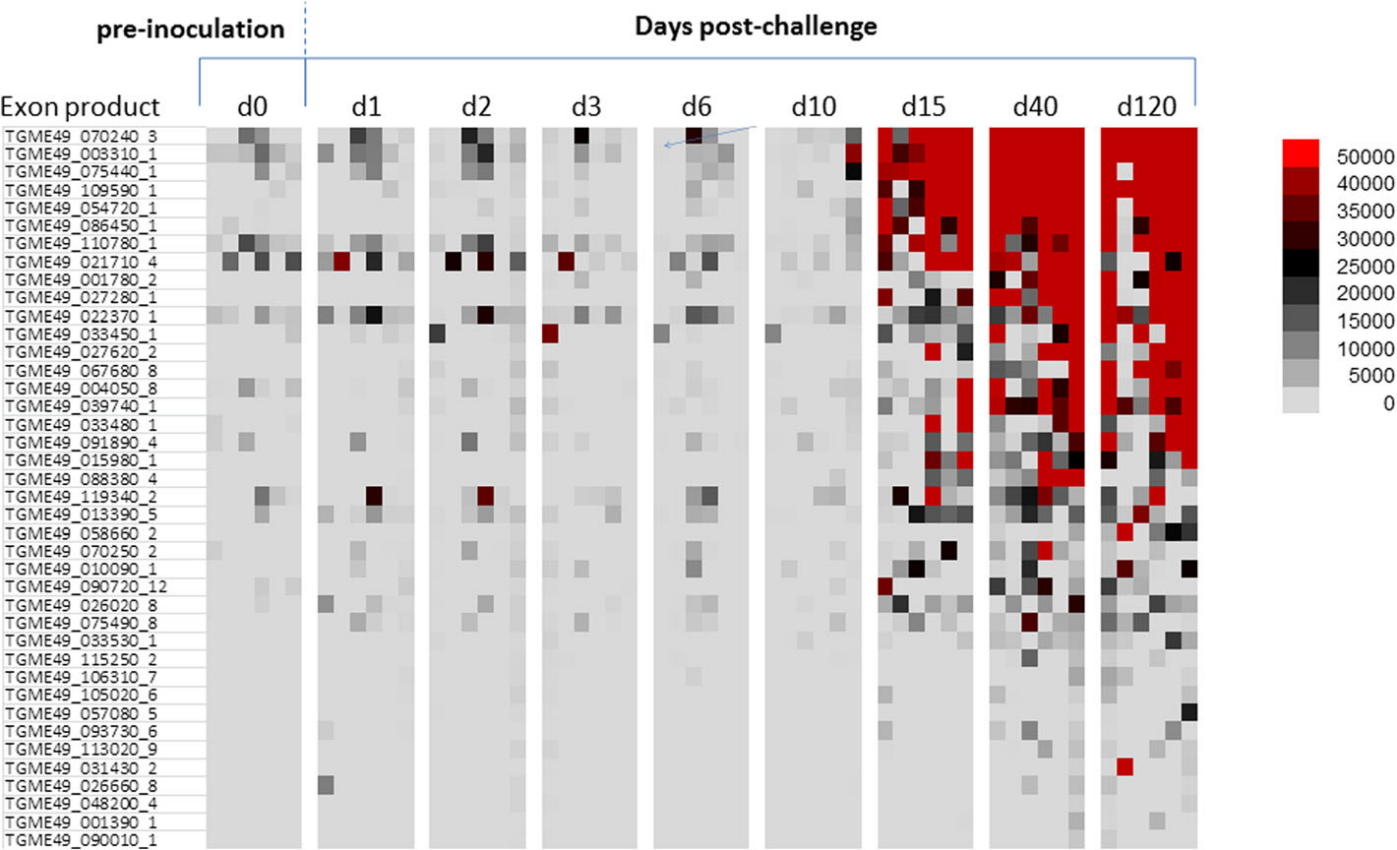
Heat map representing IgG profiles at different time points in mice inoculated orally with tissue cysts. According to the heat map, 40 antigens showed significant increase at day 40 post-infection compared to day 0 pre-inoculation values

**Fig. 8:**
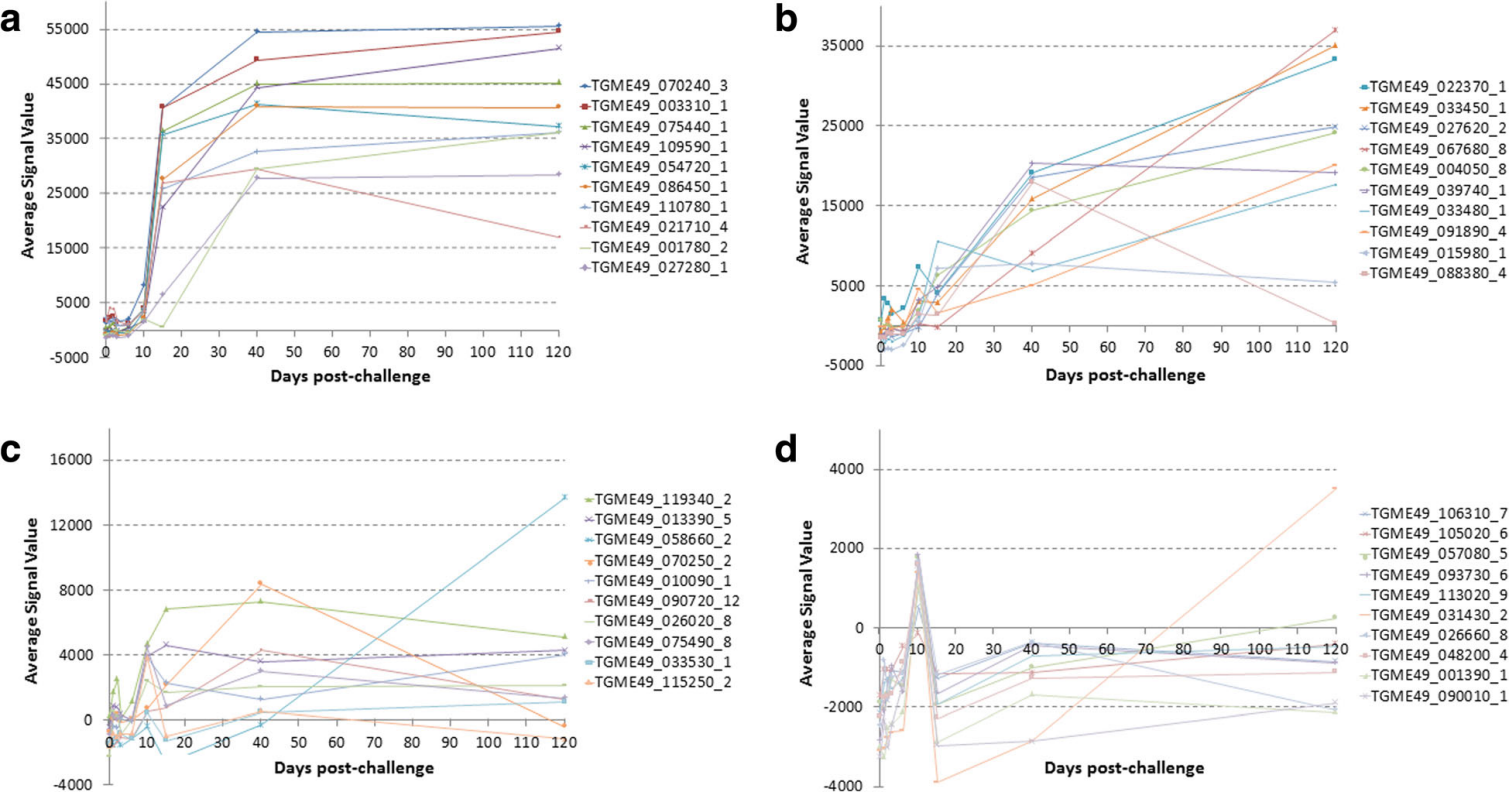
Line charts illustrate the IgG kinetics of 40 different antigens probed with sera collected serially at different time points from mice inoculated orally with tissue cysts. The Benjamini-Hochberg-corrected *P*-values of these 40 antigens comparing signal data for day 40 post-infection with day 0 pre-inoculation are also shown in Table 4. Line charts of 40 antigens are shown in four consecutive panels to increase the visibility (**a**-**d**). Signal values are average of adjusted array signals (raw IVTT signals subtracted of median of sample-specific control IVTT spots) ± SD

During the screening, IgG response against 40 antigens reached maximal intensity and breadth around day 40 as seen in the heat map in Fig. 7. IgG responses against 40 antigens increased significantly at day 40 compared to day compared to day 0 (*P*-values are listed in Table 4). The IgG response mostly gave first peaks at day 10 or afterwards (Fig. 8a-d). Among the first five highest signal giving proteins, one of them is rhoptry protein and three of them are dense granule proteins which were ESA (Table 4).

**Table 4:** Antigens that showed significant IgG response at day 40 in mice infected with tissue cysts

Rank ^a^	Gene ID_exon	Description	Benjamini-Hochberg corrected *P*-value (d0 *vs* d40)
1	TGME49_070240_3	Unspecified product (MAG1)	*P* = 0.0006
2	TGME49_003310_1	Dense granule protein GRA7	*P* = 0.0017
3	TGME49_075440_1	Granule antigen protein GRA6	*P* = 0.0006
4	TGME49_109590_1	Rhoptry protein 1	*P* = 0.0006
5	TGME49_054720_1	Dense granule protein GRA8	*P* = 0.0006
6	TGME49_086450_1	Dense granule protein 5 precursor	*P* = 0.0006
7	TGME49_110780_1	Dense granule protein GRA4	*P* = 0.0092
8	TGME49_021710_4	TBC domain-containing protein	*P* = 0.0180
9	TGME49_001780_2	Microneme protein MIC2	*P* = 0.0037
10	TGME49_027280_1	Dense granule protein GRA3	*P* = 0.0006
11	TGME49_022370_1	SAG-related sequence SRS13	*P* = 0.0187
12	TGME49_033450_1	SAG-related sequence SRS29A	*P* = 0.0475
13	TGME49_027620_2	Dense granule protein GRA2	*P* = 0.0092
14	TGME49_067680_8	Microneme protein MIC12	*P* = 0.0187
15	TGME49_004050_8	Subtilisin SUB1	*P* = 0.0317
16	TGME49_039740_1	Dense granule protein GRA14	*P* = 0.0006
17	TGME49_033480_1	SAG-related sequence SRS29C	*P* = 0.0477
18	TGME49_091890_4	Microneme protein MIC1	*P* = 0.0354
19	TGME49_015980_1	Hypothetical protein	*P* = 0.0010
20	TGME49_088380_4	Heat shock protein HSP90	*P* = 0.0012
21	TGME49_119340_2	Hypothetical protein	*P* = 0.0280
22	TGME49_013390_5	Plectin, putative	*P* = 0.0370
23	TGME49_058660_2	Rhoptry protein ROP6	*P* = 0.0355
24	TGME49_070250_2	Dense granule protein GRA1	*P* = 0.0388
25	TGME49_010090_1	Protein kinase domain-containing protein	*P* = 0.0354
26	TGME49_090720_12	Vacuolar proton translocating ATPase subunit, putative	*P* = 0.0297
27	TGME49_026020_8	Transporter, major facilitator family protein	*P* = 0.0485
28	TGME49_075490_8	Hypothetical protein	*P* = 0.0388
29	TGME49_033530_1	Hypothetical protein	*P* = 0.0099
30	TGME49_115250_2	GAMM1 protein, putative	*P* = 0.0485
31	TGME49_106310_7	RecF/RecN/SMC N terminal domain-containing protein	*P* = 0.0187
32	TGME49_105020_6	Hypothetical protein	*P* = 0.0387
33	TGME49_057080_5	3'5'-cyclic nucleotide phosphodiesterase domain-containing protein	*P* = 0.0408
34	TGME49_093730_6	DHHC zinc finger domain-containing protein	*P* = 0.0108
35	TGME49_113020_9	STAS domain-containing protein	*P* = 0.0134
36	TGME49_031430_2	Oligosaccharyl transferase stt3 protein, putative	*P* = 0.0365
37	TGME49_026660_8	Hypothetical protein	*P* = 0.0187
38	TGME49_048200_4	Ribosomal RNA- dimethyltransferase, putative	*P* = 0.0488
39	TGME49_001390_1	Hypothetical protein	*P* = 0.0180
40	TGME49_090010_1	Hypothetical protein	*P* = 0.0485

^a^Based on average signal value, as per Figs. 7 and 8

## Discussion

The realization of the importance of antigen discovery to develop diagnostic assays or a protective vaccine has increased in the last decade. During the development of a diagnostic serological assay to detect acute toxoplasmosis, antigens expressed during the acute toxoplasmosis can be much preferred. Plenty of antigens such as SAG1, SAG2, SAG2A, ROP1, ROP2, GRA1, GRA2, GRA3, GRA4, GRA5, GRA6, GRA7, GRA8, MIC1, MIC2, MIC3, MIC4, M2AP, AMA1, HSP20, BAG1 (HSP30), MAG1, NTPase, P25, P35, P68 and multi-epitope peptide containing epitopes of SAG1, SAG2 and SAG3 have been tested by ELISA methods with well categorized sera obtained from human or animal models so far to diagnose acute toxoplasmosis [13].

In the case of development of a protective vaccine against toxoplasmosis, vaccine formulations should contain antigens from each stage of the parasite to sequentially block the parasite during the invasion of host cells. More than 60 proteins have been tested as vaccine candidate antigens up to date [12], but none of them conferred the desired efficacy to be able to proceed to clinical trial. Randomly chosen vaccine candidate antigens, such as structural proteins or metabolic enzymes, have delayed the progress in vaccine development pipeline for *T. gondii*.

In the life-cycle of *T. gondii*, tissue cysts (containing bradyzoites) and oocysts (containing sporozoites) are the main forms of the parasite that transmit the disease in nature. As they enter the host cell, bradyzoites and sporozoites both convert to motile tachyzoites which invade the tissues. Just as the immune response starts to respond to the parasite, tachyzoites change into slowly dividing bradyzoites and remain latent in a tissue cyst [21]. Thus, a successful vaccine against *T. gondii* is likely to contain antigens from all forms of the parasite and selected using a rational approach.

To address these issues, we screened the serum samples from mice orally infected with tissue cysts and oocysts. Sera were collected prior to administration (Day 0) as well as 1, 2, 3, 6, 10, 15, 40 and 120 days after infection from these mice to characterize the immune response during the first dates and thereafter. By screening these mice sera, we identified the antigens that give prominent immune response during infection initiated with oocysts or tissue cysts. In addition, antibody kinetics of each antigen was displayed.

In the mice infected with oocysts, 6 IgM and 40 IgG immune-reactive antigens were discovered that showed significant increment in response compared to baseline (Figs. 1, 2, 3 and 4). In the group infected with tissue cysts, 6 IgM and 40 IgG immune-reactive antigens were identified (Figs. 5, 6, 7 and 8). Oocyst and tissue cyst fed mice showed significant IgM response at day 10. Thereafter, a decline was observed in IgM response after day 10 in oocyst-infected mice but in the group of mice infected with tissue cyst, the increment in IgM response continued until day15 and then declined. This is consistent with tissue cyst bradyzoites requiring more time to invade the host cells. The IgG response first appeared mostly around at day 15, peaked at day 40 and remained elevated or increased through day 120. Overall, there are 12 antigens discovered during IgM screening and 80 proteins during IgG screening.

If we look from the point of view of selecting an antigen(s) to develop a serological diagnostic assays or vaccine, we have to focus on the antigens that play significant roles in pathogenesis. During the invasion, MIC proteins are secreted from small apical organelles called microneme. MICs are adhesin proteins that help the parasite to attach to the host plasma membrane. ROP proteins are secreted from *unique* elongated organelles called rhoptry. ROPs are secreted from the apical end into the cytoplasm of host cells and help the formation of parasitophorous vacuole (PV) derived from host cell membrane. Once the parasite gets inside PV, GRAs which are dense granule proteins are released into the PV that modifies the vacuole membrane [22]. Thus, antigens to be used in diagnostic serological assays or vaccine formulations are needed to: (i) actively induce strong immune response at the very beginning of the infection (i.e. strong IgM response during the first 10–15 days of infection); (ii) induce long-lasting immunity (i.e. strong IgG response until day 120); (iii) be antigenic in both forms of the parasite because transmission can occur through oocysts or tissue cysts.

From the accumulated screening data, two antigens GRA6 (TGME49_075440) and GRA8 (TGME49_054720) have all of the above-mentioned properties. In addition, GRA3 (TGME49_027280), GRA5 (TGME49_086450), ROP1 (TGME49_109590) and SAG-related sequence SRS29C (TGME49_033480) have similar properties excluding antigenicity in IgM response in oocyst infected mice. Besides, there are 11 antigens that induce long-lasting IgG response in both oocyst and tissue cyst infected mice which are the cyst matrix protein MAG1 (TGME49_070240); dense granule proteins GRA2 (TGME49_027620), GRA4 (TGME49_110780), GRA7 (TGME49_003310) and GRA14 (TGME49_039740); microneme proteins MIC1 (TGME49_091890), MIC2 (TGME49_001780) and MIC12 (TGME49_067680); surface proteins SAG-related sequence SRS13 (TGME49_022370) and SRS29A (TGME49_033450); rhoptry protein ROP6 (TGME49_058660).

When we match the human screening data from our previous studies [15, 16] with these mouse response antigens, 16 of these antigens showed a strong response in human sera samples except SAG-related sequence SRS29C (TGME49_033480).

Two studies about transcriptomic and proteomic analyses of *T. gondii* confirmed that GRA2, GRA3, GRA4, GRA5, GRA6, GRA7, GRA8, GRA14, MIC1, MIC2, MIC12, ROP1, ROP6 and SRS29C, expression levels are elevated in sporozoites at days 4 and 10 of infection relative to day 0 of infection, suggesting an important role in host cell invasion [23, 24]. Among the above-mentioned antigens, ROP1, GRA2, GRA4, GRA5, GRA6, GRA7, GRA14, MIC1, MIC2 and MAG1 have been used as vaccine candidate antigens in previous studies and showed strong immunogenicity [12, 25–44]. GRA3 and GRA8 have been evaluated with ELISA and showed some immunity with human samples [45–48]. SRS29C have been identified as an important negative regulator of acute virulence in murine models [49].

## Conclusion

Our approach has revealed that ROP6, MIC12, SRS29A and SRS13 have shown strong immunogenicity and at the same time not tested in development of a diagnostic assay or a vaccine model yet. In addition, it seems that GRA3 and GRA8 can be a good vaccine candidate that has not been tested in an animal model yet. The protective efficiency and immunity of SRS29C can be analyzed in a comprehensive vaccine study. In development of a diagnostic assay to detect acute toxoplasmosis, the evaluation of ROP6, MIC12, SRS29A and SRS13 IgM/IgG kinetics with well categorized human sera can give some clue about acute toxoplasmosis. Multivalent vaccine formulations or ELISAs using several antigens of *T. gondii* have been found more successful in previous studies in terms of protection against toxoplasmosis and diagnosing acute toxoplasmosis. Thus, blending the novel antigens discovered in this study with previously immunogenic antigens in diagnostic assays or vaccine formulations may give better results. We must also bear in mind that the protection against toxoplasmosis is conferred mainly by cellular immune response and through humoral immune response [22]. Screening of cellular immunity of humans and animals against toxoplasmosis will definitely give more clues about protective antigens but a robust high-throughput wet lab approach does not exist currently. On the other hand, the antigens discovered in this study are reactive with IgM and IgG antibodies and showed strong immunogenicity and could give researchers some clue to develop a vaccine against toxoplasmosis or diagnostic serological kit.

## Additional file

Additional file 1: **Table S1.** 240 exon products reactive with murine sera comprising the mouse array TG4. (XLSX 20 kb)

## Abbreviations

ROP: rhoptry protein
GRA: dense granule protein
MIC: microneme protein
MAG: cyst matrix protein
SRS: SAG-related sequence
AVMA: American Veterinary Medical Association
FEDIAF: The European Pet Food Industry Federation
TG1, TG2/3, TG4: microarrays developed to detect *T. gondii* antigens
ToxoDB: *Toxoplasma* Genomic Resource
TGME49: *T. gondii* ME49 strain
IVTT: *in vitro* transcription/translation
SD: Standard deviation
