# Author Correction: Discovery of new *Toxoplasma gondii* antigenic proteins using a high throughput protein microarray approach screening sera of murine model infected orally with oocysts and tissue cysts

**DOI:** 10.1186/s13071-024-06322-5

**Published:** 2024-05-30

**Authors:** Mert Döşkaya, Li Liang, Aarti Jain, Hüseyin Can, Sultan Gülçe İz, Philip Louis Felgner, Aysu Değirmenci Döşkaya, David Huw Davies, Adnan Yüksel Gürüz

**Affiliations:** 1 Department of Parasitology, Vaccine Research and Development Laboratory, Faculty of Medicine, Ege University, Bornova/İzmir, Turkey; 2 Department of Medicine, Division of Infectious Diseases, University of California Irvine, Irvine, CA USA; 3 Department of Molecular Biology, Ege University Faculty of Sciences, Bornova/İzmir, Turkey; 4 Department of Bioengineering, Ege University Faculty of Engineering, Bornova/İzmir, Turkey

**Author Correction: Parasites & Vectors (2018) 11:393** 10.1186/s13071-018-2934-1

After publication [1], it was pointed out that the animal serum samples used in this study were also analyzed in a previous study under ethical approval number 2009–155 and this study was previously published online.^1^ According to Turkish legislation, the use of the same samples in different studies does not require another ethical approval. Since the authors did not specify this clearly, it appeared that the study had been done again. As a result, it was decided that providing this information would be useful for the readers.

## Footnotes

1 Döşkaya M, Caner A, Can H, Gülçe İz S, Gedik Y, Döşkaya AD, et al. (2014) Diagnostic Value of a Rec-ELISA Using *Toxoplasma gondii* Recombinant SporoSAG, BAG1, and GRA1 Proteins in Murine Models Infected Orally with Tissue Cysts and Oocysts. PLoS ONE 9(9): e108329. 10.1371/journal.pone.0108329
